# Development and Validation of a Predictive Model for Wheezing Illness Following Human Bocavirus 1 Infection in Children

**DOI:** 10.3390/microorganisms14071464

**Published:** 2026-07-03

**Authors:** Ri De, Zeng Li, Kexiang Zhang, Yao Yao, Dongmei Chen, Yu Sun, Liping Jia, Xiaolin Ma, Chunmei Zhu, Linqing Zhao

**Affiliations:** 1 Laboratory of Virology, Capital Center for Children’s Health, Capital Institute of Pediatrics, Capital Medical University, Beijing 100020, China; graceride@163.com (R.D.); drlzeng@163.com (Z.L.); 18800198237@163.com (K.Z.); xinongyaoyao@163.com (Y.Y.); dongmei_c@126.com (D.C.); sunyu780312@163.com (Y.S.); im_jiaping@126.com (L.J.); 2 Department of Respiratory Medicine, Capital Center for Children’s Health, Capital Medical University, Beijing 100020, China; pediamaxl@outlook.com

**Keywords:** children, wheezing illness, human bocavirus genotype 1, clinical predictors

## Abstract

Human Bocavirus 1 (HBoV1) is one major pathogen that has been associated with wheezing illnesses. However, there is still a lack of effective clinical predictive indicators for wheezing illnesses in children infected with HBoV1. A retrospective cohort study was conducted among pediatric patients with single-HBoV1 infection from September 2016 to August 2023. Then, univariate logistic regression was used to screen potential predictors for wheezing illness, and Least Absolute Shrinkage and Selection Operator (LASSO) regression was applied to minimize overfitting and select key predictors. Finally, a multivariate logistic regression model was constructed in a training dataset comprising 80% of patients and validated in an independent test dataset comprising 20% of patients. Then, its performance was evaluated using the Area Under the Curve (AUC). A total of 330 pediatric patients were enrolled, including 228 in the wheezing-illness group and 102 in the non-wheezing group. Three independent predictors, including abnormal NK cell percentage (OR = 1.101, 95 %CI 1.03–1.27), preterm birth (OR = 1.65, 95 %CI 1.49–1.82) and personal history of allergy (OR = 1.25, 95 %CI 1.11–1.41), were identified. The model achieved AUCs of 0.904 and 0.876 in the training and test sets, respectively. Using a Youden-derived threshold (0.382), the high-risk group in the test set had an observed wheezing rate of 85.3%, compared with 18.7% in the low-risk group (*p* < 0.001). Calibration was satisfactory (Hosmer–Lemeshow *p* = 0.324 and 0.576). A validated predictive model incorporating abnormal NK cell percentage, preterm birth and personal history of allergy accurately stratifies the risk of wheezing illness after HBoV1 infection in children, facilitating early clinical intervention.

## 1. Introduction

Human bocavirus 1 (HBoV1) is a small, non-enveloped DNA virus belonging to the genus *Bocaparvovirus* within the family *Parvoviridae*. It has emerged as a common viral pathogen of acute respiratory infections (ARIs) in children worldwide [1]. Globally, 2–20% of pediatric ARIs are associated with HBoV1. A national surveillance study in China (2009–2019) reported that HBoV1 accounted for 4.6% of all ARI patients, 6.6% of pediatric ARI cases, and 6.9% of pediatric pneumonia cases [2]. Clinical manifestations range from mild upper respiratory symptoms to severe lower respiratory tract disease, including cough, rhinitis, fever, dyspnea, and wheezing, often accompanied by diarrhea or acute otitis media [3]. Children younger than 2 years of age are the most susceptible population to HBoV1 infection [4].

Among all clinical manifestations, wheezing represents the most prominent and clinically impactful feature of HBoV1 infection [5,6]. Wheezing is defined as a continuous, high-pitched sound caused by turbulent airflow passing through narrowed lower airways. In pediatric patients, wheezing illnesses have heterogeneous etiologies and distinct phenotypes [7]. However, this is a common clinical syndrome characterized by frequent exhalation or occasional inspiratory wheezing in children infected with HBoV1 [8,9,10]. It has been revealed that HBoV1 ranks as the fourth most common respiratory virus following respiratory syncytial virus (RSV), adenovirus, and rhinovirus in children under 2 years hospitalized for acute wheezing [11].

Growing evidence indicates that primary HBoV1 infection increases the risk of persistent wheezing and even asthma in later childhood [12,13]. A follow-up study of infants hospitalized for HBoV1 bronchiolitis found that all patients developed recurrent wheezing, half were diagnosed with asthma by age 5–7 years, and nearly 30% required re-admission for wheezing exacerbations [14]. High viral loads are associated with prolonged wheezing in severe cases [15]. However, not all HBoV1-infected children develop wheezing illness, highlighting the urgent need to identify reliable predictors for early risk stratification and targeted intervention.

To address this unmet clinical need, this study systematically identified independent predictors for wheezing illness following HBoV1 infection in children in a retrospective cohort design, and developed and validated a Least Absolute Shrinkage and Selection Operator (LASSO)-based prediction model with high discriminative performance using a rigorous stepwise statistical approach, which minimized overfitting and selected key predictors. This study provides evidence-based insights into the pathogenesis of wheezing illness following HBoV1 infection in children, which may offer a tool to guide precision management and help improve long-term respiratory outcomes in susceptible children.

## 2. Materials and Methods

### 2.1. Study Design and Participants

A retrospective cohort study was conducted from September 2016 to August 2023. Pediatric patients were enrolled according to the inclusion criteria: ① visited the Capital Center for Children’s Health, Capital Medical University; ② met the World Health Organization (WHO) and the National Institute for Health and Care Excellence (NICE) diagnostic criteria for acute respiratory infection (ARIs) [16]; ③ positive for single HBoV1-DNA determined by capillary electrophoresis-based multiplex polymerase chain reaction (CEMP) assay; ④ aged 0–16 years; and ⑤ with detailed clinical information. Pediatric patients were excluded according to the exclusion criteria: ① specimens collected more than 48 h after admission; ② re-admitted within 7 days; ③ co-infected with other common respiratory pathogens determined by CEMP assay; and ④ with incomplete clinical data.

In the CEMP assay (Ningbo HEALTH Gene Technologies Ltd., Ningbo, China), a multiplex PCR was performed using 15 pairs of primers targeting 13 pathogens, human DNA, and human RNA, followed by capillary electrophoresis analysis on a GeXP capillary electrophoresis system (Sciex, Concord, ON, Canada) according to the manufacturer’s instructions. By comparing with the migration time of the size standard, the various lengths of PCR products were determined and specific pathogens were detected, as well as human DNA and human RNA: influenza virus A (Flu A) 105 nt (2009H1N1 163.3 nt, H3N2 244.9 nt), Flu B 212.7 nt, human adenovirus (AdV) 110.2/113.9 nt (representing different subtypes), HBoV 121.6 nt, human rhinovirus (HRV) 129.6 nt, human parainfluenza virus (PIV) 181.6 nt, chlamydia (Ch) 190.5 nt, human metapneumovirus (hMPV) 202.8 nt, *Mycoplasma pneumoniae* (Mp) 217 nt, human coronavirus (HCoV) 265.1 nt, and human respiratory syncytial virus (RSV) 280.3 nt [17,18].

### 2.2. Data Collection and Grouping

Clinical data were extracted from electronic medical records of patients meeting the inclusion criteria, including ① demographic characteristics of age, gender, preterm birth, and low birth weight; ② clinical symptoms of fever, rhinorrhea, cough, wheezing, chest retractions, sputum, rash, and others; ③ abnormal laboratory tests for white blood cell, neutrophil, lymphocyte counts/percentages, C-reactive protein (CRP), liver enzymes (ALT, AST), lactate dehydrogenase (LDH), creatine kinase (CK, CK-MB), adenosine deaminase (ADA), and plasma bicarbonate; ④ abnormal immunological markers for serum total immunoglobulin E (IgE), eosinophil count, lymphocyte subsets (CD4+, CD8+, CD4+/CD8+ ratio, T-cell %, B-cell %, NK cell %), and immunoglobulins IgA, IgG, and IgM; ⑤ radiographic findings of chest X-ray (X-ray) or Computed Tomography (CT); ⑥ comorbidities and history findings of personal history of allergy, family allergy history, and any recorded complications during hospitalization; and ⑦ treatment strategies, such as auxiliary ventilation, antibiotic use, and length of hospital stay. The data were preprocessed before analysis. All investigations were ordered by attending physicians based on clinical judgment and standard protocols, rather than being mandated by the study protocol. Not all patients underwent every investigation; the availability of each data element reflects routine clinical practice. Chest CT was performed only when clinically indicated.

Patients enrolled in the study were divided into wheezing or non-wheezing groups. Wheezing was defined as a repetitive, high-pitched, exhalation-oriented sound caused by turbulence in the narrow lower airways, and the wheezing sound of upper airway noises and exercise-induced wheezing were excluded according to the Global Strategy for Asthma Management and Prevention [19]. Patients who were positive only for HBoV1 and had symptoms of wheezing in ARIs or a diagnosis of asthma/asthma exacerbation or acute bronchitis were assigned to the wheezing group, while patients with wheezing symptoms of other diseases, such as foreign body aspiration, bronchopulmonary dysplasia, and cystic fibrosis, that could explain the wheezing episodes, were excluded. Patients with a confirmed single HBoV1 infection and no documented wheezing during the illness episode were assigned to the non-wheezing group.

### 2.3. Sample Size and Data-Splitting

To ensure the representativeness of the training and validation subsets, the entire dataset was partitioned using stratified random sampling based on the wheezing illness outcome. Each group was randomly allocated into two sets, with 80% of patients assigned to the training set for model development and the remaining 20% reserved as an independent test set for performance evaluation. A fixed random seed was set to ensure the reproducibility of the data split. The training set was used for predictor selection and model fitting, while the test set was held out for internal validation to assess the generalizability of the final predictive model.

### 2.4. Univariate Logistic Regression

Univariate logistic regression was performed to identify individual associations between each candidate variable and wheezing illness. Variables with a *p* value < 0.05 in the univariate analysis were selected for inclusion in the subsequent LASSO regression.

### 2.5. LASSO Regression

To mitigate multicollinearity and overfitting, LASSO regression was applied to variables selected through univariate analysis. The optimal penalty parameter λ was determined via 10-fold cross-validation; λ.min was chosen over λ.1se to prioritize predictive performance and retain clinically relevant predictors. Variables with non-zero coefficients at λ.min were entered into the final multivariate logistic regression model. The cross-validation curve is shown in Supplementary Figure S1.

### 2.6. Multivariate Logistic Regression

Using the variables selected by LASSO, a multivariate logistic regression model was fitted on the training set. Odds ratios (ORs) with 95 % confidence intervals (CIs) were reported for each predictor in the final model.

### 2.7. Model Performance

The discriminative performance of the model was assessed using the Area Under the Receiver Operating Characteristic Curve (AUC). An AUC of below 0.5 indicates no discrimination, 0.7–0.8 is considered acceptable, 0.8–0.9 is excellent, and >0.9 is outstanding. Model performance was evaluated on both the training and the test sets. Calibration of the predicted probabilities was examined using the Hosmer–Lemeshow goodness-of-fit test.

### 2.8. Statistical Analyses

All statistical analyses were performed using R software (version 4.5.2; R Foundation for Statistical Computing) with relevant packages (glmnet for LASSO, pROC for ROC analysis). A two-sided *p* value < 0.05 was considered statistically significant.

## 3. Results

### 3.1. Baseline Characteristics of the Study Cohort

From September 2016 to August 2023, a total of 16,397 respiratory tract specimens were collected. A total of 3017 were excluded for not being collected within 48 h of admission, and 1011 for not being the first obtained during a single hospitalization or for being from children re-admitted within 7 days of discharge. Among the remaining 12,369 specimens, 778 were positive for HBoV1 determined by CEMP assay, including 399 co-positive for other pathogens and 379 only positive for HBoV1. Then, 29 cases were excluded due to incomplete clinical data, and 20 were excluded due to diagnoses, such as leukemia, trauma, etc., not associated with respiratory infections. Therefore, 330 patients mono-positive for HBoV1 were included to search for predictors of wheezing illness.

Then, the detailed clinical features of the 330 patients, including demographic characteristics, clinical symptoms, abnormal laboratory results, abnormal immunological markers, abnormal radiographic findings, comorbidities, history data, and treatment strategies, were collected. According to the Global Strategy for Asthma Management and Prevention, 228 patients with a median age of 1.81 (IQR: 1.21, 2.88) years, a male-to-female ratio of 1.92:1, and wheezing illness constituted the wheezing illness group, while the remaining 102 patients with a median age of 1.93 (IQR: 1.03, 3.46) years, a male-to-female ratio of 1.91:1, and no wheezing illness formed the non-wheezing group (Supplemental Table S1).

### 3.2. Identification of Candidate Predictors via Univariate Logistic Regression

Univariate logistic regression analysis was performed to screen for potential risk factors of wheezing illness associated with single HBoV1 infection. As detailed in Table 1, 25 variables demonstrated a statistically significant risk association (*p* < 0.05) with HBoV1-related wheezing illness. Among these, the candidate predictors (OR > 1) included preterm birth (OR = 2.54, 95 % CI: 1.24–5.76), personal history of allergy (OR = 1.01, 95 % CI: 1.00–1.03), chest retractions (OR = 2.91, 95 % CI: 1.21–7.12), abnormal eosinophil count (OR = 2.80, 95 % CI: 1.25–6.52), CD8+ T-lymphocyte percentage (OR = 1.11, 95 % CI: 1.07–1.16), CD4+ T-lymphocyte percentage (OR = 1.10, 95 % CI: 1.06–1.13), T-lymphocyte percentage (OR = 1.10, 95 % CI: 1.07–1.13), NK cell percentage (OR = 1.09, 95 % CI: 1.02–1.15), IgG (OR = 1.18, 95 % CI: 1.07–1.30), IgA (OR: 2.05, 95 % CI: 1.32–3.40), and lymphocyte percentage (OR = 9.51, 95 % CI: 2.79–33.71) (Table 1).

### 3.3. Variables Selected Using LASSO Regression

**Figure 1:**
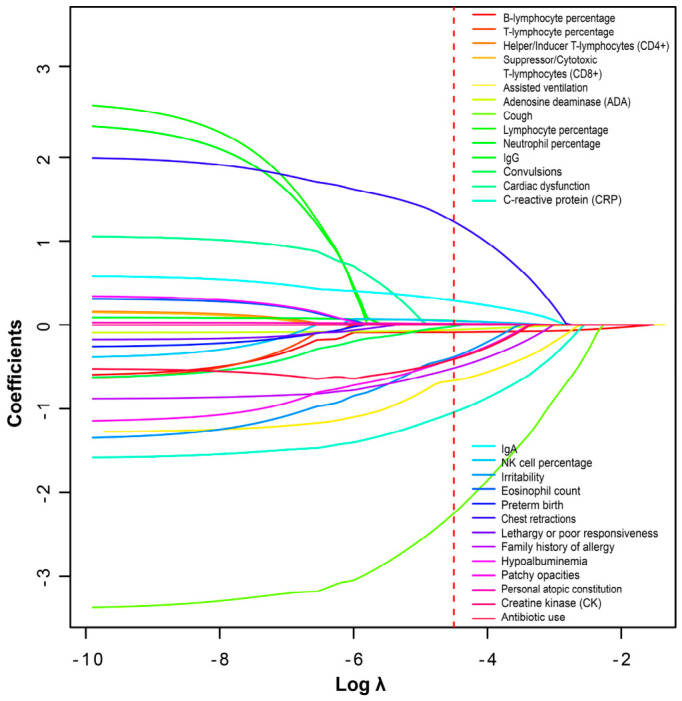
**LASSO coefficient paths for predictor selection.** This plot visualizes the shrinkage of regression coefficients as the penalty parameter (Log(λ)) increases. Each colored line represents the trajectory of a specific clinical or laboratory variable. The numbers across the top axis indicate the number of non-zero variables remaining in the model at that specific λ.

To refine the candidate variables and prevent model overfitting, LASSO regression was applied to the significant predictors from the univariate analysis. The cross-validation curve (Supplemental Figure S1) indicated the optimal lambda (λ) value, which corresponded to the most parsimonious model with minimal deviance. The coefficient profile plots (Figure 1) illustrated how the coefficients of the predictors shrank as the penalty increased. At the optimal λ, LASSO regression selected 15 non-zero coefficient variables for the subsequent multivariate model (Table 2), and the intercept represented the expected log-odds of the outcome when all predictors were zero.

### 3.4. Multivariate Logistic Regression Model and Independent Predictor

The 15 variables selected by LASSO were entered into a multivariate logistic regression model (Table 3). After adjusting for all other variables in the model, three factors retained independent statistical significance as predictors of wheezing illness. The abnormal NK cell percentage (OR = 1.11, 95 %CI 1.03–1.27), preterm birth (OR = 1.65, 95 %CI 1.49–1.82), and personal history of allergy (OR = 1.25, 95 %CI 1.11–1.41) emerged as independent and statistically significant predictors. The variables of cough, antibiotic use, assisted ventilation, family history of allergy, abnormal IgA, patchy opacities, abnormal IgG, irritability, and convulsions were not statistically significant in the multivariate model (all *p* > 0.05) (Table 3).

### 3.5. Scoring System

**Figure 2:**
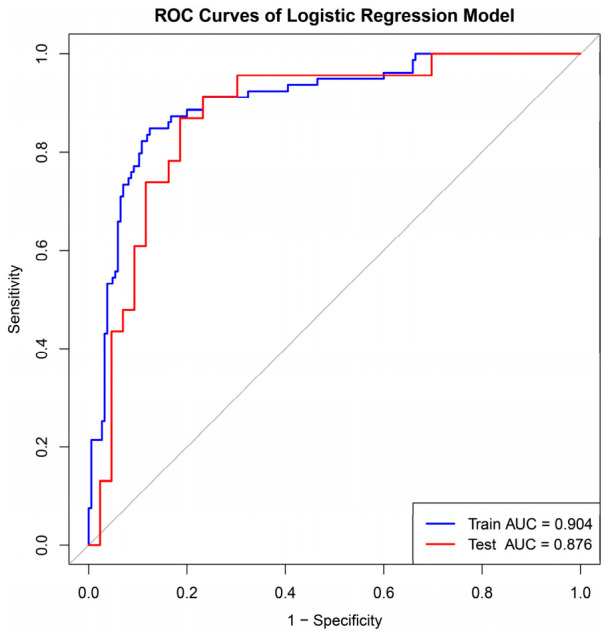
**ROC curves of the logistic regression model for training and test sets.** Receiver Operating Characteristic (ROC) curves illustrate the diagnostic performance of the multivariate logistic regression model. The blue line represents the training set, with an AUC of 0.904. The red line represents the independent test set, showing an AUC of 0.876. The *x*-axis represents the false positive rate (1–specificity) and the *y*-axis represents the true positive rate (sensitivity). The diagonal gray line represents the performance of a random classifier (AUC = 0.5).

The discriminative performance of the final multivariate logistic regression model was evaluated by the AUC. On the training set, the AUC was 0.904. When applied to the independent test set, the model maintained good performance with an AUC of 0.876 (Figure 2).

**Figure 3:**
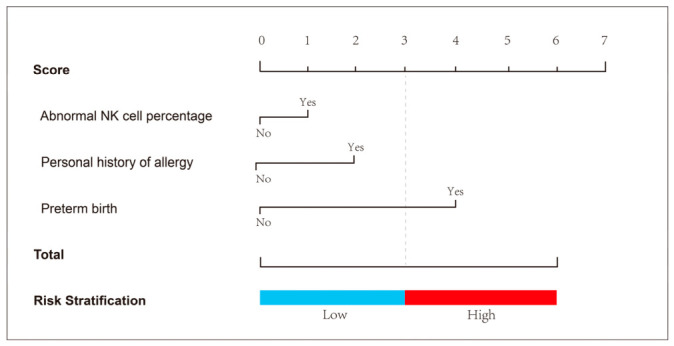
**Simplified clinical scoring tool for early identification of children at risk of HBoV1-induced wheezing.** This nomogram-style scoring chart translates the three independent predictors (abnormal NK cell percentage, personal history of allergy, and preterm birth) into an integer point system. Points are assigned as follows: abnormal NK cell percentage (present = 1 point), personal history of allergy (present = 2 points), and preterm birth (present = 4 points). The total score ranges from 0 to 7. Based on the Youden index-derived cutoff (dash line) of ≥3 points from the training set, patients are stratified into low-risk (total score 0–2) and high-risk (total score 3–7) groups. In the independent test set, the high-risk group had a wheezing rate of 84.2%, compared with 19.5% in the low-risk group (*p* < 0.001). The simplified scoring system achieved a sensitivity of 0.81 and a specificity of 0.79 for predicting post-HBoV1 wheezing illness.

To facilitate bedside risk estimation, a simplified integer scoring system based on the β-coefficients of the three independent predictors was derived. Points for the other predictors were calculated proportionally and rounded to the nearest integer. The total score ranged from 0 to 7 (Figure 3).

In the training set, the Youden index identified a cutoff of ≥3 points for high-risk classification (sensitivity 0.81, specificity 0.79). In the test set, the wheezing rate was 84.2% in the high-risk group versus 19.5% in the low-risk group *(p* < 0.001), confirming the clinical utility of this simplified scoring system. The optimal predicted probability threshold determined by the Youden index in the training set was 0.38, corresponding to a sensitivity of 0.81, a specificity of 0.79, and a Youden index of 0.61. Applying this threshold to the independent test set yielded a significantly higher wheezing rate in the high-risk group (85.32%) than in the low-risk group (18.65%; *p* < 0.001, χ^2^ test), supporting the practical value of this risk stratification. Calibration was satisfactory, as the Hosmer–Lemeshow test showed no significant lack of fit (training set *p* = 0.32; test set *p* = 0.58).

## 4. Discussion

This study searched for the risk predictors of wheezing illness following HBoV1 infection in children, and then a predictive model was developed. Through a comprehensive analytical approach incorporating univariate screening, LASSO regression for variable selection, and multivariate logistic regression, three independent predictors, abnormal NK cell percentage, preterm birth, and personal history of allergy, were significantly associated with wheezing illness in HBoV1 infections. The predictive model incorporating these factors achieved excellent discriminative ability, with AUC values of 0.904 and 0.876 in the training and test sets, respectively.

Preterm birth was identified as an independent predictor of wheezing illness in HBoV1 infections, consistent with robust epidemiological evidence. A meta-analysis of over 1.5 million children has demonstrated a 46% increased risk of childhood wheezing disorders associated with preterm birth, with even higher risks for very preterm infants (<33 weeks) [20]. Several mechanisms may explain this association. First, preterm infants have structurally and functionally immature lungs, characterized by fewer, larger alveoli and thickened septa, which reduce pulmonary reserve and increase susceptibility to airflow limitation during acute infection. Second, the premature lung is particularly vulnerable to early viral infections that can disrupt alveolarization and promote long-term airway hyperreactivity [21]. Third, relative immaturity of both innate and adaptive immunity in preterm infants delays viral clearance and exacerbates airway inflammation. Fourth, preterm birth often coexists with bronchopulmonary dysplasia (BPD), a well-established risk factor for recurrent wheezing. Notably, wheezing in preterm children is typically non-atopic and poorly responsive to inhaled corticosteroids, suggesting that HBoV1-induced wheezing in this population arises predominantly from structural airway vulnerability and impaired innate antiviral defense rather than classical allergic sensitization.

Abnormal NK cell percentage was identified as an independent predictor of wheezing following HBoV1 infection, underscoring the role of innate immune dysfunction in disease pathogenesis. NK cells provide first-line antiviral defense through cytolysis and secretion of immune-regulatory cytokines, notably IFN-γ. However, HBoV1 has evolved mechanisms to evade innate immunity that may specifically compromise NK cell function. The viral nonstructural protein NP1 inhibits the type I interferon pathway by blocking IRF-3, thereby suppressing IFN-β production and impairing NK cell activation. Children with low NK cell proportions at presentation may thus have limited capacity to overcome viral evasion, leading to delayed clearance, persistent airway inflammation, and increased wheezing risk. The contribution of NK cells to wheezing following viral infection is virus-dependent and context-sensitive. In RSV infection, NK cells are indispensable for viral control but can exacerbate lung injury via IFN-γ, exhibiting a dual role [22]. During HBoV1 infection, the virus may dysregulate NK cell recruitment and function, transforming them from protective effectors into promoters of aberrant inflammation. Moreover, NK cells can either promote type-2 immunity and airway hyper-responsiveness or suppress it, depending on the time of infection relative to allergic sensitization [23]. Collectively, these observations suggest that abnormal NK cell percentage in HBoV1-infected children reflects a state of broader innate immune dysregulation, where compromised NK function contributes both to impaired viral control and to enhanced airway inflammation.

Personal history of allergy demonstrated a modest but statistically significant association with wheezing illness following HBoV1 infection. This finding highlights the importance of host immune predisposition in determining clinical outcomes after viral infection. Atopic constitution, characterized by a genetic tendency to produce immunoglobulin E (IgE) in response to environmental allergens, reflects a Th2-polarized immune milieu. In atopic individuals, respiratory viral infections can synergize with allergic inflammation to promote wheezing through multiple mechanisms. First, viral infection of the airway epithelium in atopic hosts may amplify Th2-type inflammatory responses, leading to enhanced eosinophil recruitment and activation [24]. Second, the disrupted epithelial barrier during viral infection facilitates allergen penetration and presentation, potentially triggering allergic inflammation that coexists with and exacerbates viral-induced airway narrowing [25]. Third, viral infections can induce IgE production specific to viral antigens, contributing to mast cell degranulation and immediate-type hypersensitivity reactions in the airways [26]. The interplay between viral infection and atopic predisposition is particularly relevant in the context of HBoV1, as studies have demonstrated that children with atopic traits exhibit prolonged viral shedding and more severe lower respiratory tract symptoms [27].

Regarding disease severity as an important dimension that may influence the risk of subsequent wheezing, we have collected all variables related to the severity of disease in this study, such as respiratory failure, hypoxemia, acid–base imbalance, electrolyte imbalance, coagulation dysfunction, cardiac dysfunction, liver dysfunction, gastrointestinal dysfunction, consciousness change, and assisted ventilation. Through the initial univariate screening, only assisted ventilation entered the LASSO selection process, and showed no independent statistical significance in the final multivariable model. This suggested that the three predictors of wheezing illness, including percentage of NK cells, preterm birth, and allergy history, had nothing to do with the severity of acute infection of HBoV1 in infants and young children.

Moreover, it is important to acknowledge that the contribution of each predictor may vary across age groups [28]. Prematurity-related structural airway deficits are most relevant in early childhood, when lung growth and alveolarization are still ongoing, while in adolescents, the impact of premature birth on airway function may be attenuated by compensatory lung growth. Similarly, allergic sensitization—which typically evolves over the first several years of life—may not be fully manifested in young infants, yet a personal history of allergy in this age group may reflect early atopic traits that nonetheless confer increased risk [29,30]. The NK cell abnormality, by contrast, may represent a more consistent risk factor across age groups, as innate immune function is relatively stable throughout childhood. These age-dependent nuances should be considered when applying the model in clinical practice, and future studies with larger samples should specifically examine whether the predictive performance of each factor varies across age strata.

Several limitations of this study should be acknowledged. First, the retrospective design inherently predisposes to selection bias and precludes definitive causal inferences regarding the identified associations. Second, as a single-center study, the generalizability of our findings to populations with distinct genetic backgrounds or environmental exposures may be limited. Third, the pathogen-detection panel only covers 13 common pathogens. Co-infections with untested or emerging pathogens may influence outcomes. Fourth, the study period spans the COVID-19 pandemic. Non-pharmaceutical interventions may have altered the epidemiological patterns of pediatric respiratory infections. Although infection rates were lower during the pandemic, we cannot exclude potential influences from changes in healthcare, viral transmission, or population immunity.

## 5. Conclusions

This study revealed that abnormal NK cell percentage, preterm birth, and personal history of allergy were significant independent predictors for the development of wheezing illness in children following HBoV1 infection. The predictive model established herein offers a reliable and easy-to-operate tool for early identification of patients at high risk for wheezing illness (total score ≥3 points), so that more active monitoring or treatment can be provided to these children to prevent recurrent wheezing or asthma.

## Figures and Tables

**Table 1:** Univariate logistic regression analysis of predictors for HBoV1-related wheezing illness.

Independent Variables	Coefficient (β)	*p* Value	Odds Ratio (OR)	95 % Confidence Interval (CI)
Preterm birth	0.93	0.02	2.54	1.24–5.76
Convulsions	−1.01	<0.001	0.37	0.19–0.65
Cough	−2.8	<0.001	0.06	0.01–0.23
Irritability	−1.61	<0.001	0.2	0.05–0.58
Eosinophil count	1.03	0.01	2.8	1.25–6.52
Suppressor/Cytotoxic T-lymphocytes (CD8+)	0.1	<0.001	1.11	1.07–1.16
Helper/Inducer T-lymphocytes (CD4+)	0.09	<0.001	1.1	1.06–1.13
T-lymphocyte percentage	0.09	<0.0001	1.1	1.07–1.13
B-lymphocyte percentage	−0.12	<0.001	0.89	0.86–0.91
NK cell percentage	0.08	<0.001	1.09	1.02–1.15
IgG	0.16	<0.001	1.18	1.07–1.30
IgA	0.72	<0.001	2.05	1.32–3.40
Personal history of allergy	0.01	0.02	1.01	1.00–1.67
Creatine kinase (CK)	≤0.001	0.02	1	0.99–1.00
Adenosine deaminase (ADA)	−0.09	<0.001	0.92	0.87–0.96
Neutrophil percentage	−1.98	<0.001	0.14	0.04–0.41
Lymphocyte percentage	2.25	<0.001	9.51	2.79–33.71
Patchy opacities	−1.27	0.02	0.28	0.08–0.74
Family history of allergy	−0.66	0.02	0.52	0.29–0.89
Cardiac dysfunction	−1.12	<0.001	0.33	0.16–0.62
Hypoalbuminemia	−1.45	0.02	0.24	0.06–0.69
Chest retractions	1.07	0.02	2.91	1.21–7.12
C-reactive protein (CRP)	−1.04	<0.001	0.35	0.17–0.68
Assisted ventilation	−1.45	<0.001	0.24	0.12–0.43
Antibiotic use	−0.82	0.04	0.44	0.20–0.96

**Table 2:** LASSO regression analysis for variable selection.

Category	Coefficient	Category	Coefficient
Intercept *	12.12	Abnormal IgA	0.29
Abnormal B-lymphocyte percentage	−0.09	Abnormal NK cell percentage	0.05
Assisted ventilation	−0.64	Convulsions	−0.38
Abnormal adenosine deaminase (ADA)	−0.06	Preterm birth	1.14
Cough	−2.25	Family history of allergy	−0.52
Abnormal IgG	0.05	Patchy opacities	−0.40
Antibiotic use	−0.41	Personal history of allergy	0.02
Abnormal C-reactive protein (CRP)	−1.04	Irritability	−0.41

Note: * the expected log-odds of the outcome when all predictors were zero.

**Table 3:** Multivariate logistic regression analysis of predictive factors.

Category	Coefficient (β)	Standard Error (SE)	z Statistic	*p* Value	Odds Ratio (OR)	95 %Confidence Interval (CI)
Abnormal B-lymphocyte percentage	−0.10	0.02	−4.69	0.00	0.90	0.86–0.94
Abnormal adenosine deaminase (ADA)	−0.15	0.04	−3.31	0.00	0.86	0.79–0.94
Abnormal NK cell percentage	0.13	0.05	2.12	0.05	1.11	1.03–1.22
Preterm birth	0.52	0.05	10.36	<0.0001	1.65	1.49–1.82
Personal history of allergy	0.22	0.06	4.04	<0.0001	1.25	1.11–1.41
Cough	−2.62	1.35	−1.94	0.05	0.07	0.00–0.74
Abnormal C-reactive protein (CRP)	−1.18	0.58	−2.03	0.04	0.31	0.09–0.92
Antibiotic use	−1.35	0.70	−1.93	0.05	0.26	0.06–0.99
Assisted ventilation	−1.02	0.53	−1.91	0.06	0.36	0.12–0.99
Family history of allergy	−0.71	0.48	−1.50	0.13	0.49	0.18–1.20
Abnormal IgA	0.34	0.32	1.07	0.29	1.41	0.85–2.66
Patchy opacities	−0.87	0.87	−1.00	0.32	0.42	0.06–2.09
Abnormal IgG	0.07	0.09	0.74	0.46	1.07	0.89–1.29
Irritability	−0.63	0.92	−0.69	0.49	0.53	0.07–2.71
Convulsions	−0.04	0.52	−0.08	0.94	0.96	0.34–2.63

## Data Availability

The data presented in this study are available on request from the corresponding author.

## Supplementary Materials

The following supporting information can be downloaded at: https://www.mdpi.com/article/10.3390/microorganisms14071464/s1, Figure S1. Cross-Validation for Penalty Parameter (Log(λ)) Selection. The relationship between model error (Binomial Deviance) and the penalty parameter (Log(λ)) is shown. Red dots represent the mean binomial deviance for each λ, with vertical gray error bars indicating the standard error. The vertical red dashed line on the left corresponds to λmin, the value of λ that results in the lowest cross-validation error. The vertical blue dashed line on the right represents λ1se, the largest λ within one standard error of the minimum. For this study, λmin was utilized to ensure the inclusion of all independently contributing predictors. Supplementary Table S1. Demographic characteristics of HBoV1-related wheezing illness group and non-wheezing illness group.
