# Supplementary material for: Development and Validation of a Predictive Model for Wheezing Illness Following Human Bocavirus 1 Infection in Children
Source: Microorganisms. 2026 Jul 3;14(7):1464. doi: 10.3390/microorganisms14071464 (PMC13413856; doi:10.3390/microorganisms14071464)
Supplement: Supplementary file 1 [file microorganisms-14-01464-s001.zip › Supplemental Figure S1 Cross-Validation for Penalty Parameter (Log(λ)) Selection.pdf]

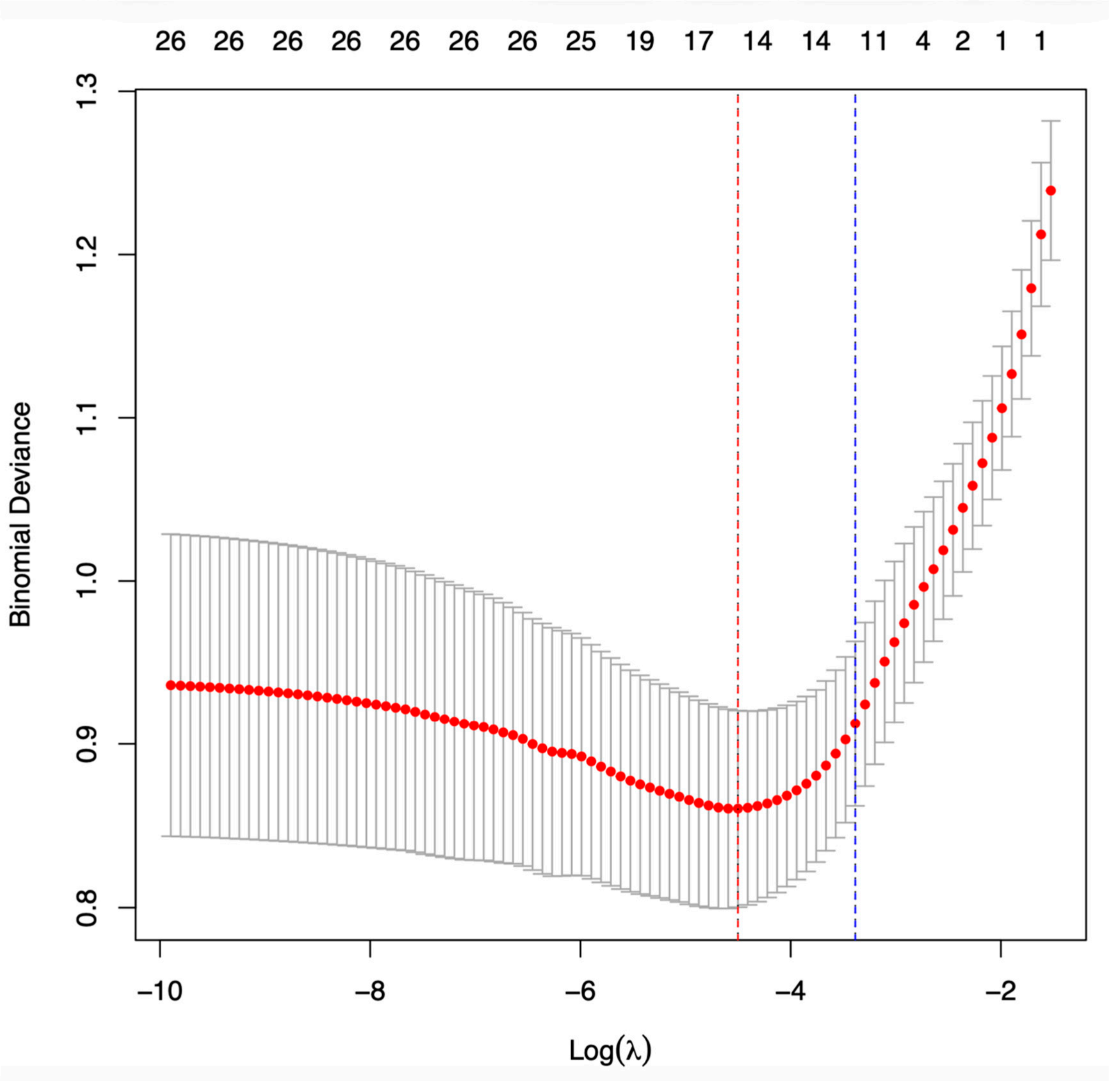

Supplementary Figure S1. Cross-Validation for Penalty Parameter (Log(λ)) Selection. The relationship between model error (Binomial Deviance) and the penalty parameter (Log(λ)) is shown. Red dots represent the mean binomial deviance for each **λ**, with vertical gray error bars indicating the standard error. The vertical red dashed line on the left corresponds to **λmin**, the value of **λ** that results in the lowest cross-validation error. The vertical blue dashed line on the right represents **λ1se**, the largest **λ** within one standard error of the minimum. For this study, **λmin** was utilized to ensure the inclusion of all independently contributing predictors.
